# Prevalence of Hirschsprung-associated enterocolitis in patients with Hirschsprung disease

**DOI:** 10.1007/s00383-021-05020-y

**Published:** 2021-09-30

**Authors:** J. Hagens, K. Reinshagen, C. Tomuschat

**Affiliations:** Department of Paediatric Surgery, Kinder-UKE Campus Ost 45, University Medical Center Hamburg-Eppendorf, Martinistrasse 52, 20246 Hamburg, Germany

**Keywords:** Hirschsprung Disease, Hirschsprung-associated enterocolitis, HAEC, Prevalence, Preoperative, Postoperative

## Abstract

**Purpose:**

Hirschsprung's associated enterocolitis (HAEC) is a complication of Hirschsprung's Disease (HD) with considerable morbidity and mortality. The variability in presentation leads to a wide variety of the reported prevalence pre-and postoperatively. This systematic review aimed to clarify the prevalence of HAEC in short—(S-HD), long (L-HD), TCA and the type of operation used.

**Methods:**

A systematic literature-based search for relevant cohorts was performed using Pubmed/Medline, Cochrane Library from its inception to May 2021. Studies reporting on pre-and postoperative enterocolitis, segment length, and surgical procedure (Soave, Swenson, Duhamel) were included. Pooled prevalence and subgroup analysis have been calculated for pre-and postoperative HAEC.

**Results:**

4738 articles were identified from the literature search, among which 57 studies, including 9744 preoperative and 8568 postoperative patients, were included. The groups were sorted by length of the aganglionic segment for further analysis. The pooled prevalence for preoperative HAEC was 18.3% for all types, 15.2% for S-HD and 26.1% for TCA. The pooled prevalence for postoperative HAEC was in total 18.2% for all segment lengths and used techniques. Subgroup analysis showed no significant difference in the occurrence of postoperative enterocolitis between the three techniques.

**Conclusion:**

The prevalence of preoperative HAEC increases with segment length. However, pooled data suggest that the postoperative risk for developing HAEC, independently of the employed method and segment length, is comparable to the preoperative risk.

## Introduction

Hirschsprung's Disease (HD) is a developmental disorder of the enteric nervous system (ENS) with an estimated incidence of 1:5000 newborns. Because of the failure of migration of neural crest cells, patients lack ganglion cells in the distal bowel and with the earlier migration arrest, the longer the aganglionic segment [1]. One of the major complications in the treatment of HD is Hirschsprung's associated enterocolitis (HAEC), which is responsible for the most severe morbidity and mortality in HD [2]. The main culprit in our understanding of the aetiology of HAEC is the variable diagnostic criteria used for reporting incidence. The symptoms exhibited in HAEC are often nonspecific, and a high index of suspicion is usually required for diagnosis. HAEC is a clinical condition characterised by explosive diarrhoea, abdominal distension, colicky abdominal pain, lethargy, and fever. The spectrum ranges from mild abdominal distension, with loose stools and perianal excoriation, to life-threatening toxic megacolon with explosive diarrhoea, vomiting, rectal bleeding, lethargy and eventually shock and death [3–5]. It is essential to notice that HAEC can occur pre- and postoperatively.

The reported incidence of HAEC varies significantly in published series and ranges from 17 to 50%. Preoperative HAEC has been reported to occur in 5.7–50%. Also, the incidence correlates with the length of the aganglionic segment, being highest in patients with total colonic aganglionosis (TCA) [6]. The incidence of postoperative colitis has been reported to occur in 2–35% of patients [3]. Postoperative HAEC can occur > 18 months after definitive surgery. However, most patients have their last episode of significant HAEC within 2 years of their pull-through procedure. Several studies have shown that some patients who develop HAEC are prone to recurrent episodes. The incidence of recurrent HAEC varies from 5.2 to 56% and may be attributed to an underlying immunological defect that leads to chronic, relapsed gut inflammation [3, 7]. Several case series suggest an association between HD and inflammatory bowel disease (IBD) [8]. Since HAEC occurs postoperatively in a significantly high percentage, several studies have addressed the type of operation (Soave, Swenson, and Duhamel) as a single risk factor for HAEC, with conflicting results [9–11]. Up to date, no operation is superior to the other in preventing HAEC. Because of the wide variety of the reported prevalence pre-and postoperatively, this systematic review aimed to clarify the prevalence of HAEC in short—(S-HD), long (L-HD) and TCA, and the type of operation used. The study's findings will help to clarify conflicting results reported in the current literature regarding the prevalence of HAEC in HD. Further, the exact prevalence will help facilitate the design of evidence-based preoperative management and follow-up and better care for patients with Hirschsprung's Disease.

## Methods

A systematic literature-based search for relevant cohorts was performed using PubMed/Medline, Cochrane Library, from inception to May 2021. To avoid selection bias, no filter options were used in the initial search. For PubMed, the search strategy was used as follows: search terms *(((Hirschsprung) OR (megacolon)) OR (aganglionosis)) AND* followed by different specifications: *(enterocolitis)*; *(incidence)*; *(long segment)*; *(Duhamel)*; *(Swenson)*; *(pull-through)*; *(torre)*. The strategy was adapted in line with the indexing systems of the other databases. The reference lists of all studies and systematic reviews identified were evaluated for additional relevant studies.

### Inclusion criteria

**Fig. 1:**
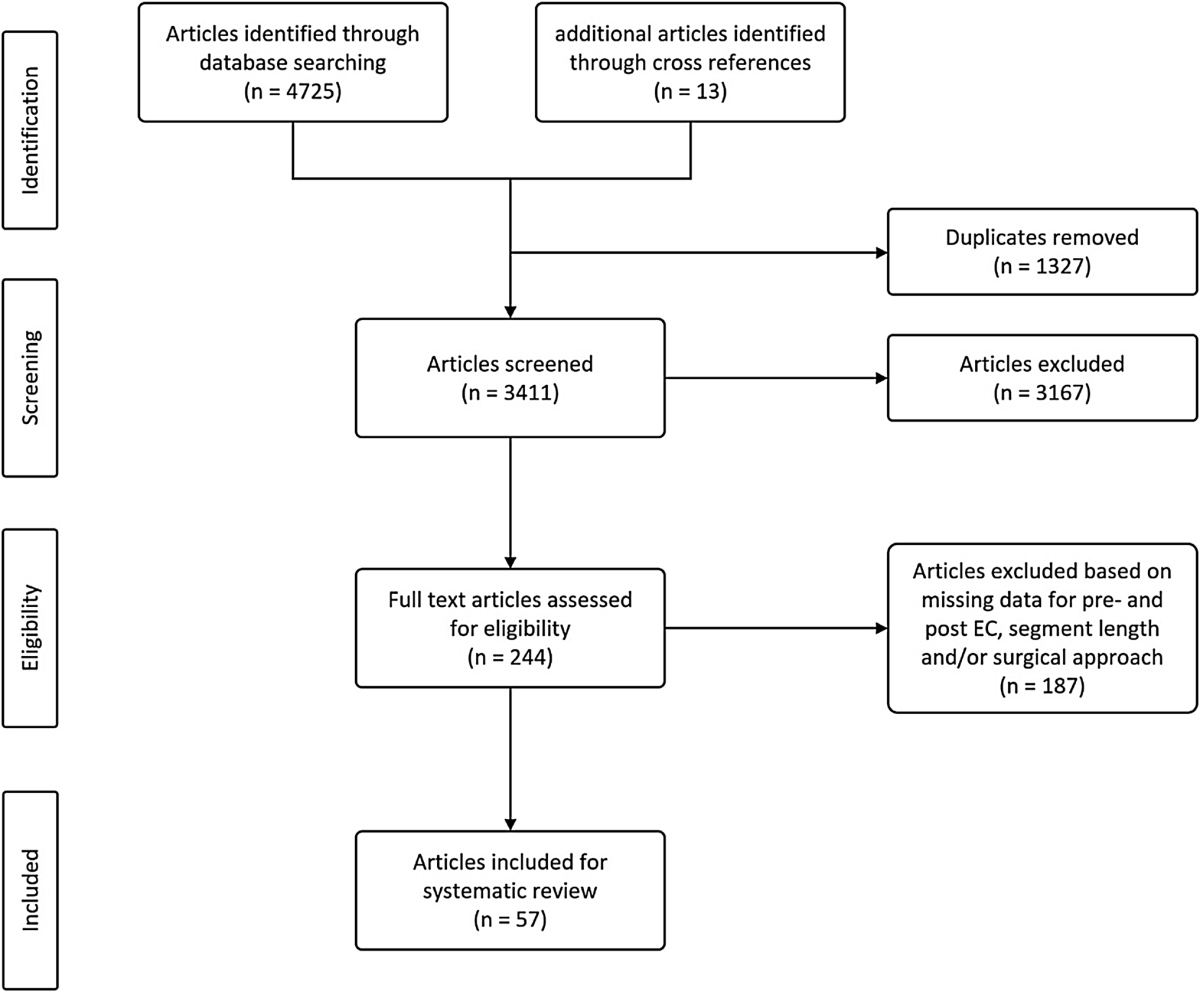
PRISMA flow chart of the screening process

After removal of duplicates, all titles and abstracts were scanned in an initial screening. Original studies reporting Hirschsprung's associated enterocolitis (HAEC) were included and reviewed for the following inclusion criteria: (1) data available for both pre-and postoperative HAEC; (2) data given for the length of the aganglionic segment; (3) data on the surgical approach (Duhamel, Swenson, Soave, including their modifications). Exclusion criteria were: (1) non-accessible papers due to non-English language or no full-text availability; (2) systematic reviews and meta-analyses without original data from the authors; (3) studies conducted on animals only or focussing on molecular biological or pathological mechanisms of HD or HAEC; (4) no data on both pre-and postoperative HAEC; (5) undefined aganglionic segment length; (6) undefined surgical approach or no surgery for Hirschsprung's disease. After the initial screening, 244 original full-text articles were identified and reviewed for their eligibility for inclusion into the analysis. The complete screening process is listed in the PRISMA flowchart (see Fig. 1).

### Data extraction

The data from all eligible studies were recorded in a standardised spreadsheet that included key characteristics of the studies, such as year of publication, geographical region, study design, number of patients, age at operation, length of the aganglionic HD segment (i.e., short, long, total colonic), surgical approaches (i.e., Swenson, Duhamel, Soave), number of stages in surgery (one stage or staged), number of pre-and postoperative EC (Table 1).

**Table 1:** Key characteristics of the included studies

Author	Year of Publication	Country	Study design	Number of patients pre-OP	S-HD	L-HD	TCA	Surgical procedure	EC pre-OP	EC post-OP	Congenital anomalies
Carcassonne et al	1989	France	Cohort study	32	22	7	2	Swenson, Soave, Duhamel	11	4	/
Carneiro et al	1992	UK	Cohort study	76	61	8	7	Swenson, Soave, Duhamel	24	7	Down' syndrome (*n* = 8), cardiac defects (*n* = 8), gastrointestinal (*n* = 4), genitourinary (*n* = 4), central nervous system (*n* = 3)
Downey et al	2015	USA	Cohort study	24	21	2	4	Soave, Duhamel	7	7	Down Syndrome (*n* = 7), Turner Syndrome (*n* = 1), cardiac defects (*n* = 4), central hypoventilation syndrome (*n* = 1), esophageal atresia (*n* = 1), duodenal atresia (*n* = 1), omphalocele (*n* = 1), renal duplication (*n* = 1)
Elhalaby et al	1995	USA	Cohort study	168	124	27	10	Swenson, Soave, Duhamel	21	44	Down' Syndrome (*n* = 16), other (*n* = 31)
Fortuna et al	1996	USA	Cohort study	82	66	11	5	Soave, Duhamel	10	20	Down' Syndrome (*n* = 7)
Foster et al	1990	USA	Cohort study	63	44	5	9	Swenson, Soave, Duhamel	6	4	/
Harrison et al	1986	USA	Cohort study	139	95	21	14	Swenson, Soave, Duhamel, Myectomy	23	13	Down's Syndrome (*n* = 14), cleft lip and palate (*n* = 3), supernumary digits (*n* = 3), congenital hip dislocation (*n* = 2), congenital heart disease (*n* = 2), mental retardation (*n* = 2), microcephalus (*n* = 1), hydrocephalus (*n* = 1), growth hormone deficiency (*n* = 1), deafness (*n* = 1), facial nerve palsy (*n* = 1), microphthalmus (*n* = 1), brachial cleft cyst (*n* = 1), duplication of small bowel (*n* = 1), sucrase or maltase deficiency (*n* = 1), agenesis of the left kidney (*n* = 1), imperforate anus type II (*n* = 1), undescended testis (*n* = 1), bilateral tallpes equinovarus (*n* = 1), Legg-Calvé-Syndrome (*n* = 1), Ichthyosis congenita (*n* = 1), Siemens' Syndrome (*n* = 1)
Ikeda and Goto	1984	Japan	Nationwide study	1628	1239	186	137	Swenson, Soave, Duhamel	475	180	Down's Syndrome (*n* = 47), cardiac anomalies (*n* = 41), inguinal hernia (*n* = 11), malrotation (*n* = 10), cleft lip and palate (*n* = 9), Meckel's diverticulum (*n* = 8), mental retardation (*n* = 8), poly- or syndactyria (*n* = 6), hydrocephalus (*n* = 6), atresia ani (*n* = 5), megaureter (*n* = 2), others (*n* = 80)
Jung	1995	Korea	Cohort study	137	90	19	4	Duhamel	35	12	genitourinary anomalies (*n* = 15), Down's Syndrome (*n* = 2), cardiac defects (*n* = 5), gastrointestinal anomalies (*n* = 7), gross malformation of face and skull (*n* = 5), skeletal anomalies (*n* = 3), others (*n* = 7)
Kleinhaus et al	1979	USA	Nationwide study	1196	299	609	90	Swenson, Soave, Duhamel	179	99	/
Le-Nguyen et al	2019	Canada	Case–control study	171	130	21	14	Swenson, Soave, Duhamel, Myectomy	25	33	Down's Syndrome (*n* = 9), Mowat-Wilson-Syndrome (*n* = 2)
Li et al	2006	China	Cohort study	252	147	4	1	Soave	20	34	/
Menezes and Puri	2006	Ireland	Cohort study	259	209	50	Swenson, Soave, Duhamel, Myectomy	43	56	Down's Syndrome (*n* = 39), others (*n* = 15)	Menezes and Puri
Moore et al	1996	South Africa	Cohort study	178	123	41	14	Swenson, Soave, Duhamel	30	19	/
Neuvonen et al	2015	Finland	Cohort study	146	121	10	15	Soave	24	64	Down's Syndrome (*n* = 25), cartilage-hair hypoplasia (*n* = 6), cardiac defects (*n* = 5), Mowat-Wilson-Syndrome (*n* = 5), Haddad-Syndrome (*n* = 3), vesicoureteral reflux (*n* = 3), anorectal malformation (*n* = 2), hypertelorism (*n* = 2), undescended testis (*n* = 1), pyloric stenosis (*n* = 1), choanal atresia (*n* = 1), cleft palare (*n* = 1), vena Galeni malformation (*n* = 1), trigonocephalia (*n* = 1), club fett (*n* = 1), Currarino-Syndrome (*n* = 1), Waardenbourg-Syndrome (*n* = 1), marker Chromosome Syndrome (*n* = 1)
Pini Prato et al	2019	Italy	Case–control study	385	273	27	74	Soave, Duhamel	66	87	Down's Syndrome (*n* = 23), anomalies of kidney and urinary tract (*n* = 6), CHD (*n* = 8), visual impairment (*n* = 7), cerebral abnormality (*n* = 1), other (*n* = 3)
Pini Prato et al	2008	Italy	Cohort study	112	80	6	22	Swenson, Soave, Duhamel	39	25	urogenital anomalies (*n* = 9), central nervous system (*n* = 8), cardiac defects (*n* = 5), ocular anomalies (*n* = 4), Down's syndrome (*n* = 2), others (*n* = 17)
Ramesh et al	1999	Malaysia	Cohort study	40	27	10	3	Rehbein, Swenson, Duhamel, TERPT, Myectomy	5	3	Down's Syndrome (*n* = 3)
Reding et al	1997	Belgium	Cohort study	59	45	3	8	Swenson, Duhamel, Myectomy	13	17	Down's Syndrome (*n* = 7), genitourinanry anomalies (*n* = 4), cardiac defects (*n* = 3), Waardenburg-Syndrome (*n* = 1), others (*n* = 2)
Rescorla et al	1992	USA	Cohort study	260	174	61	25	Swenson, Soave, Duhamel, Myectomy	15	32	Down's Syndrome (*n* = 23)
Sauer et al	2005	Canada	Cohort study	24	19	2	3	Soave, Duhamel	7	0	/
Singh et al	2003	Australia	Nationwide study	126	76	22	7	Soave, Duhamel	15	17	Down's Syndrome (*n* = 13), cardiac defects (*n* = 2), development delayed (*n* = 2), others (*n* = 16)
Surana et al	1994	Ireland	Cohort study	135	98	25	12	Swenson, Soave, Duhamel	25	20	Down's Syndrome (*n* = 17)
Teitelbaum et al	1988	USA	Cohort study	80	68	5	7	Swenson, Soave, Duhamel, Myectomy	15	5	Down's Syndrome (*n* = 13), cardiac defects (*n* = 10), central nervous system (*n* = 6), gastrointestinal anomalies (*n* = 3), genitourinary anomalies (*n* = 2), maxillofacial anomalies (*n* = 3), skelettal anomalies (*n* = 2)
Teitelbaum et al	2000	USA	Case–control study	181	134	26	16	Soave	27	55	Down's Syndrome (*n* = 11), cardiac defects (*n* = 11), others (6)
Suita et al	1996	Japan	Nationwide study	1121	877	135	109	Swenson, Soave, Duhamel Myectomy	326	188	Down's Syndrome (*n* = 80), cardiac defects (*n* = 67), malrotation (*n* = 19), mental retardation (*n* = 10), intestinal atresia (*n* = 9), cleft lip and palate (*n* = 8), Ondine's Syndrome (*n* = 8), anomalies of the limb (*n* = 7), anorectal malformation (*n* = 6), inguinal hernia (*n* = 3), Meckel's diverticulum (*n* = 3)
Suita et al	2005	Japan	Nationwide study	1103	856	143	104	Swenson, Soave, Duhamel, TERPT, Myectomy	191	117	Down's Syndrome (*n* = 507), cardiac defects (*n* = 202), mental retardation (*n* = 49), malrotation (*n* = 29), anomalies of the limb (*n* = 21), anorectal malformation (*n* = 20), Ondine's Syndrome (*n* = 19), cleft lip and palate (*n* = 18), ingulinal hernia (*n* = 14), dwarfism (*n* = 14), microcephalus (*n* = 13), Meckel's diverticulum (*n* = 11), intestinal atresia (*n* = 9), hydrocephalus (*n* = 7), undescended testis (*n* = 7), vesico-ureteral reflux (*n* = 7)
Ali	2010	Egypt	Cohort study	28	15	6	0	Soave	7	4	Down's Syndrome (*n* = 4), cardiac defects (*n* = 3), hypospadias (*n* = 1)
Chiengkriwate et al	2007	Thailand	Case–control study	50	41	9	0	Duhamel	9	18	Down's syndrome (*n* = 2), cardiac defects (*n* = 1), cerebral palsy (*n* = 1)
Giuliani et al	2011	Italy	Cohort study	70	60	10	0	Soave, Duhamel	10	2	Down's Syndrome (*n* = 3), hypothyreodism (*n* = 1), plurimalformative syndrome (*n* = 1)
Hackham et al	2004	USA	Cohort study	66	52	14	0	Soave, Duhamel	13	17	Down's syndrome (*n* = 5), cardiac defects (*n* = 5), pulmonary defects (*n* = 4), chromosomal anomalies (*n* = 2), orthopedic anomalies (*n* = 1), diaphragmatic hernia (*n* = 1), imperforate anus (*n* = 1)
Haricharan et al	2008	USA	Cohort study	52	40	12	0	TERPT	3	19	Down's Syndrome (*n* = 4), malrotation (*n* = 1), unilateral renal agenesis (*n* = 1)
Langer et al	2003	Canada, USA, Mexico	Cohort study	141	110	7	0	Soave	20	9	Down's Syndrome (*n* = 4), cardiac defects (*n* = 3), central hypoventilation syndrome (*n* = 1)
Lin et al	2020	China	Case–control study	95	78	17	0	Soave	19	15	0
Mattioli et al	2008	Italy	Cohort study	46	38	8	0	Swenson, Soave, Duhamel	11	4	Urogenital anomalies (*n* = 5), central nervous system (*n* = 4), Down's Syndrome (*n* = 3), cardiac defects (*n* = 2), Ondine's Syndrome (*n* = 1), metabolic disorders (*n* = 1), eye malformations (*n* = 1)
Mir et al	2001	Turkey	Cohort study	10	9	1	0	Duhamel	1	2	Down's syndrome (*n* = 1), umbilical hernia (*n* = 1)
Ouladsaiad et al	2016	Morocco	Cohort study	15	13	2	0	TERPT	6	4	/
Parahita et al	2018	Indonesia	Cohort study	100	93	7	0	Soave, Duhamel	9	15	/
Singh et al	2007	Canada	Cohort study	52	46	6	0	Swenson	5	6	Down's Syndrome (*n* = 5)
Wang et al	2004	China	Cohort study	61	58	3	0	Soave	22	13	/
Wu et al	2009	China	Cohort study	97	89	8	0	Swenson	17	19	/
Zhang et al	2014	China	Cohort study	127	113	14	0	TERPT	43	2	/
Adigüzel et al	2017	Turkey	Cohort study	50	41	0	0	Soave	11	10	Down's Syndrome (*n* = 3), cardiac defects (*n* = 2), cleft palate (*n* = 1)
Chung et al	2019	China	Cohort study	96	96	0	0	Soave, Duhamel	10	20	cardiac defects (*n* = 14), Down's syndrome (*n* = 9), neurological disorders (*n* = 8), genitourinary anomalies (*n* = 6), gastrointestinal anomalies (n = 5), endocrine diseases (*n* = 6), others (*n* = 10)
Fujiwara et al	2007	Japan	Cohort study	35	35	0	0	Soave	0	7	0
Gao et al	2001	China	Cohort study	34	34	0	0	Soave	5	2	Down's Syndrome (*n* = 1), undescended testis (*n* = 1)
Langer et al	1999	USA	Cohort study	9	9	0	0	Soave	2	2	Down's Syndrome (*n* = 1)
Pratap et al	2007	Nepal	Cohort study	65	65	0	0	Soave	30	3	/
Sapin et al	2006	France	Cohort study	21	21	0	0	Swenson, Soave	1	1	central hypoventilation syndrome (*n* = 1)
Weidner and Waldhausen	2003	USA	Cohort study	15	15	0	0	Swenson	1	2	Down's Syndrome (*n* = 1)
Zakaria et al	2012	Egypt	Cohort study	40	40	0	0	Soave	9	3	/
Escobar et al	2005	USA	Cohort study	36	0	0	36	Soave, Duhamel	3	11	Ondine's Syndrome (*n* = 3), UDT (*n* = 3), Down's Syndrome (*n* = 2), cardiac defects (*n* = 2), cleft lip and palate (*n* = 3), corneal opacification (*n* = 1), hydrocephalus (*n* = 1), optic nerve hypoplasia (*n* = 1), hypospadias (*n* = 1), other (*n* = 4)
Hoehner et al	1998	Canada	Cohort study	29	0	0	29	Soave, Duhamel	1	16	Down's Syndrome (*n* = 3), colonic atresia (*n* = 2), Warden's Syndrome (*n* = 1), solitary kidney (*n* = 1)
Menezes et al	2008	Ireland, Italy	Cohort study	58	0	0	58	Swenson, Soave, Duhamel	13	31	Down's Syndrome (*n* = 2), cardiac defects (*n* = 4), renal anomalies (*n* = 4), mental retardation (*n* = 3), visual defects (*n* = 2), Ondine's Syndrome (*n* = 1)
Wildhaber et al	2005	USA	Cohort study	25	0	0	25	Swenson, Soave, Duhamel	5	11	/
Yan et al	2020	China	Cohort study	35	0	0	35	Soave	17	10	Cardiac defects (*n* = 2), Meckel's diverticulum (*n* = 1), primary immunodeficiency disease (*n* = 1)
Yeh et al	2014	China	Cohort study	9	0	0	9	Duhamel	3	5	Hypoplastic right heart syndrome (*n* = 1), imperforate anus (*n* = 1), urogenital sinus, vaginal atresia, Pallister-Hall-syndrome (*n* = 1)

### Statistical analysis

Statistical analysis was performed using the EpiGear MetaXL (version 5.3) software add-in for Microsoft Excel. For the estimation of pooled prevalence, the double arcsine transformation was used [12]. Results were calculated with a 95% CI. Expecting a high heterogeneity, a random-effects model was selected, and heterogeneity was assessed using the *I*^*2*^ statistic, which describes the percentage of variation across studies not only resulting from sampling error. An *I*^*2*^ value above 75% was defined as an indicator for high heterogeneity. Since this analysis aimed to estimate the pooled effect size without testing a hypothesis, there was no *p* *value* calculation for the pooled prevalence analysis. Subgroup analysis to compare surgical techniques was undertaken using the Cochrane RevMan5 software (version 5.4). A *p* *value* < 0.05 was defined as statistically significant for this analysis, and results were presented with a 95% CI.

## Results

### Study characteristics

In total, 4738 articles were identified from the literature search. After removing the duplicates, the titles and abstracts from 3411 articles were scanned, and 244 articles were included for full-text screening. Fifty-seven studies with a total of 9744 patients matched the inclusion criteria were analysed. Among these, 48 were cohort studies, including 5 nationwide studies, and 4 were case–control studies. The studies were implemented at various paediatric healthcare institutions from 21 countries, with the USA and China being the most common. The studies included patient data ranging from the year 1955 to the year 2018. Forty-one studies included patients with various congenital diseases or syndromes, of which Trisomy 21 was the most frequently reported. All studies focused on the surgical therapy of patients suffering from Morbus Hirschsprung. The age at operation was available for 40 studies and ranged from < 1 week to 23 years. The time to follow-up was available for 37 studies and ranged from 0 to 25 years.

### Segment length

In total, data were available on 9744 preoperative and 8568 postoperative patients, and the majority of the studies provided information for every HD segment type. For the analysis of the pooled prevalence for pre-and postoperative enterocolitis, studies were divided into the following groups: (1) studies reporting on all types of HD segments; (2) studies excluding TCA patients; (3) studies reporting on short segment HD only; (4) studies reporting only on TCA patients.

**Fig. 2:**
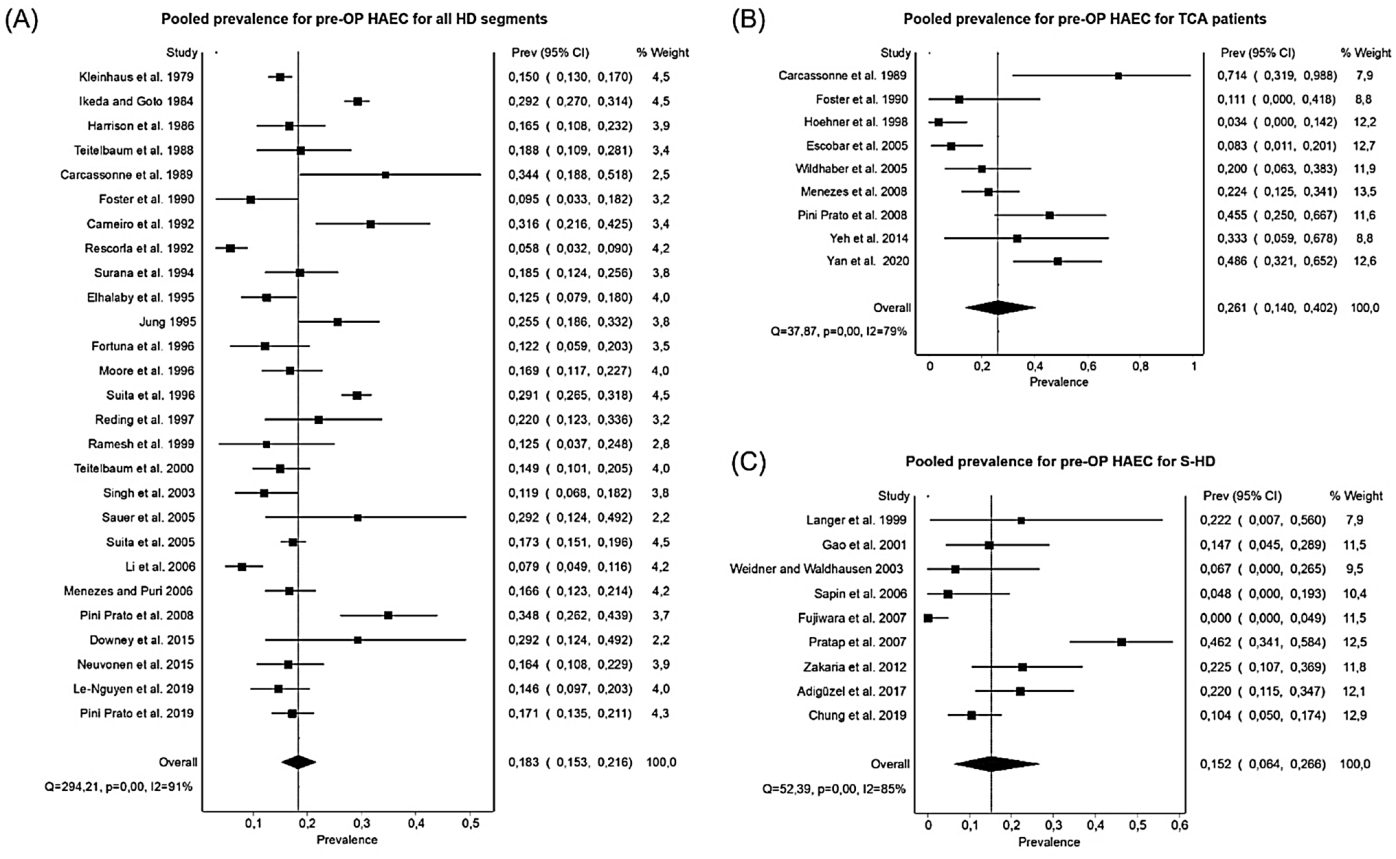
Forest Plots for the pooled prevalence of preoperative HAEC for different segment types. **A** Pooled prevalence for all HD segment types. **B** Pooled prevalence for TCA patients. **C** Pooled Prevalence for S-HD

The overall pooled prevalence for HAEC for all HD segment types and used techniques was 0.185 (95% CI 0.162–0.210, Cochrane *Q* test, *I*^2^ = 87%) for preoperative and 0.182 (95% CI 0.157–0.207, Cochrane *Q* test, *I*^2^ = 87%) for postoperative patients. Under the exclusion of patients with TCA, the overall preoperative prevalence for HAEC slightly decreased to 0.183 (95% CI 0.159–0.209, Cochrane *Q* Test, *I*^2^ = 88%). For preoperative HAEC, the pooled prevalence was 0.183 for all types of HD (*n* = 8177, 95% CI 0.153–0.216, Cochrane *Q* test, *I*^2^ = 91%), 0.191 for S-HD and L-HD (*n* = 1010, 95% CI 0.144–0.242, Cochrane *Q* test, *I*^2^ = 73%), 0.152 for S-HD only (*n* = 365, 95% CI 0.064–0.266, Cochrane *Q* test, *I*^2^ = 85%), and 0.261 for TCA only (*n* = 230, 95% CI 0.140–0.402, Cochrane *Q* test, *I*^2^ = 79%) (Fig. 2). The pooled prevalence for postoperative HAEC was 0.171 for all types of HD (*n* = 7019, 95% CI 0.144–0.199, Cochrane *Q* test, *I*^2^ = 88%), 0.159 for S-HD and L-HD (*n* = 1007, 95% CI 0.103–0.226, Cochrane *Q* test, *I*^2^ = 85%), 0.131 for S-HD only (*n* = 364, 95% CI 0.081–0.189, Cochrane *Q* test, *I*^2^ = 53%), and 0.462 for TCA only (*n* = 216, 95% CI 0.373–0.552, Cochrane *Q* test, *I*^2^ = 39%).

### Surgical procedure

Studies reporting on the same type of HD segment were used to analyse the pooled prevalence for the different surgical techniques. For the Swenson operation, no satisfactory preoperative data for HAEC was available. There were eight studies (*n* = 585) providing data for postoperative HAEC after Swenson procedure, and all of them had patients with all types of HD. The pooled prevalence for postoperative HAEC in these studies was 0.197 (95% CI 0.135–0.267, Cochrane *Q* test, *I*^2^ = 50%). There was a similar prevalence for postoperative HAEC after Duhamel operation with 0.197 (*n* = 142, 95% CI 0.032–0.436, Cochrane *Q* test, *I*^2^ = 87%), whereas the pooled prevalence for preoperative HAEC in these four studies was lower (0.158, *n* = 145, 95% CI 0.103–0.222, Cochrane *Q* test, *I*^2^ = 0%). The Soave operation showed the lowest prevalence for postoperative HAEC with only 0.114 (*n* = 635, 95% CI 0.054–0.192, Cochrane *Q* test, *I*^2^ = 85%), starting with a comparatively high preoperative HAEC prevalence of 0.195 (*n* = 635, 95% CI 0.124–0.276, Cochrane *Q* test, *I*^2^ = 82%) in the nine used studies. All studies that were used to calculate the pooled prevalence after the Duhamel and Soave procedure excluded TCA patients.

**Fig. 3:**
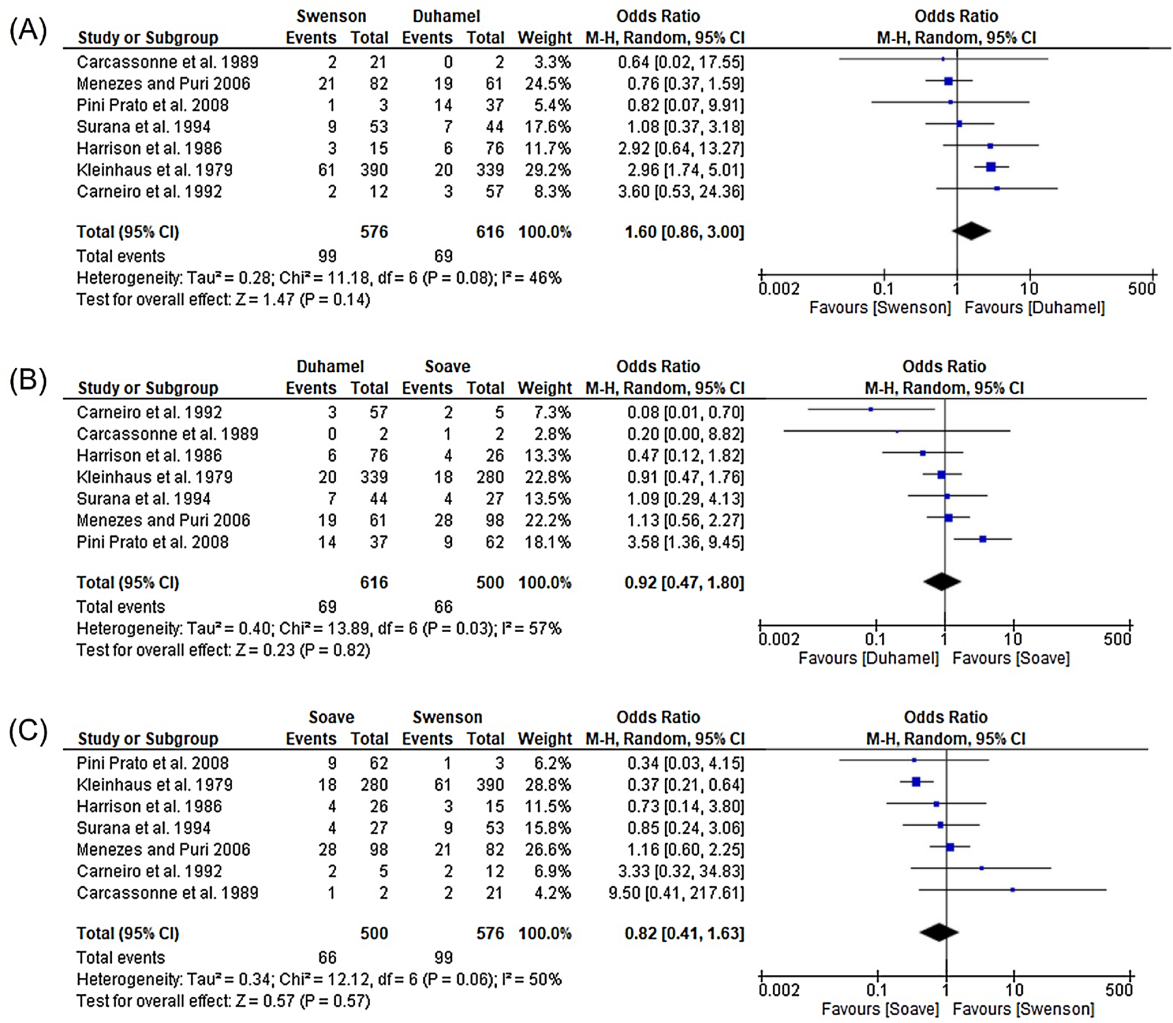
Subgroup analysis for the comparison of postoperative HAEC between different surgical procedures. **A** Comparison between Swenson and Duhamel procedure. **B** Comparison between Duhamel and Soave procedure. **C** Comparison between Soave and Swenson procedure

Seven studies (*n* = 1692) were reporting on postoperative HAEC for all three major surgical approaches. Therefore, a subgroup analysis with these studies was conducted to compare the HAEC prevalence for the Swenson, Duhamel and Soave approach (Fig. 3). Comparing the Swenson with the Duhamel technique, there was a tendency towards Duhamel (OR 1.60, 95% CI 0.86–3.00, *P* = 0.08, *I*^2^ = 46%). This trend was also visible in comparing the Duhamel and the Soave technique with an Odds Ratio of 0.92 (95% CI 0.47–1.80, *P* = 0.03, *I*^2^ = 57%). The comparison between the Soave and Swenson technique showed slightly more postoperative HAEC in the Soave group (OR 0.82, 95% CI 0.41–1.63, *P* = 0.06, *I*^2^ = 50%). Although there was a tendency towards the Duhamel approach, no significant difference in the occurrence of postoperative enterocolitis between the three techniques could be shown in the subgroup analysis.

### Surgical stages

Eleven studies, including 770 preoperative and 769 postoperative patients, provided data for pre-and postoperative HAEC regarding the number of stages used. Two of these studies included patients with all types of HD segment; six studies excluded TCA patients, and three studies reported only on S-HD. Only two studies were reporting on pre-and postoperative HAEC for multi-stage procedures. Therefore, no reliable statement on this method was possible. The pooled prevalence in one-stage pull-through operations was 0.134 (95% CI 0.080–0.199, Cochrane *Q* test, *I*^2^ = 79%) for preoperative HAEC and 0.187 (95% CI 0.121–0.264, Cochrane *Q* test, *I*^2^ = 81%) for postoperative HAEC.

### Surgical technique

**Fig. 4:**
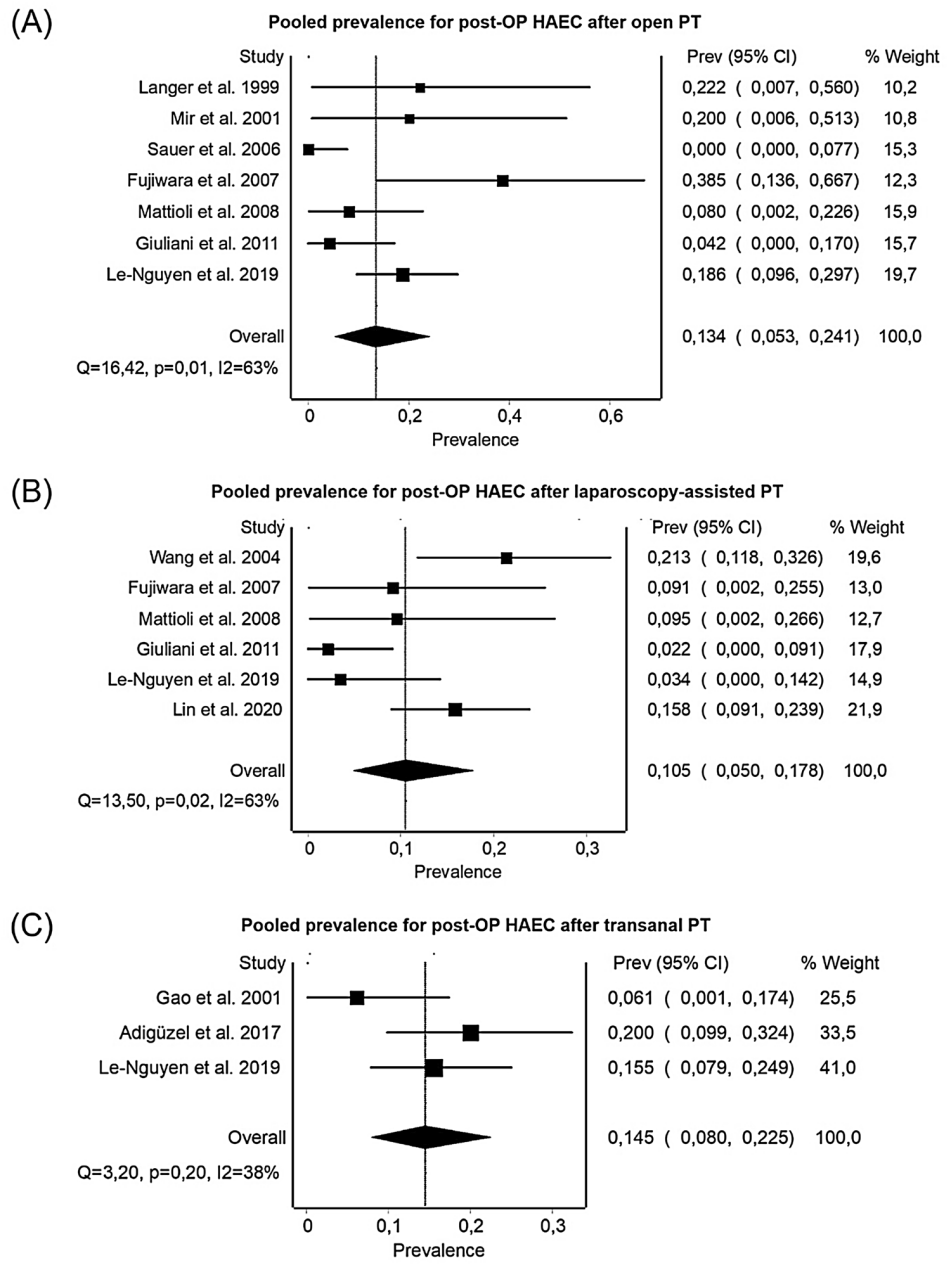
Forest Plots for the pooled prevalence of postoperative HAEC for different surgical techniques. **A** Pooled prevalence for HAEC after open pull through. **B** Pooled prevalence for HAEC after laparoscopically assisted pull through. **C** Pooled Prevalence for HAEC after totally transanal pull through

There were 11 studies, including 593 preoperative and 590 postoperative patients, which reported on both pre-and postoperative HAEC regarding the surgical technique: 3 studies provided data only for open surgery and 3 studies for open and laparoscopy-assisted procedures; 2 studies reported only on laparoscopy-assisted or only transanal pull-throughs, and one study provided data on all three types of surgery. Two of these studies included patients with all types of HD segment; five studies excluded TCA patients, and four studies reported only on S-HD. The pooled prevalence for preoperative HAEC was 0.186 (*n* = 164, 95% CI 0.117–0.265, Cochrane *Q* test, *I*^2^ = 29%) for the open surgery group, 0.190 (*n* = 274, 95% CI 0.094–0.307, Cochrane *Q* test, *I*^2^ = 78%) for the laparoscopy-assisted group and 0.177 (*n* = 155, 95% CI 0.0.121–0.241, Cochrane *Q* test, *I*^2^ = 0%) for the totally transanal group. All three approaches lead to a reduction of postoperative HAEC, with the laparoscopy-assisted procedure presenting the lowest rate in HAEC postoperatively: the pooled prevalence for postoperative HAEC was 0.134 (*n* = 162, 95% CI 0.053–0.241, Cochrane *Q* test, *I*^2^ = 63%) for the open procedure, 0.105 (*n* = 274, 95% CI 0.050–0.178, Cochrane *Q* test, *I*^2^ = 63%) for the laparoscopy-assisted group and 0.145 (*n* = 154, 95% CI 0.080–0.225, Cochrane *Q* test, *I*^2^ = 38%) for the transanal approach (Fig. 4).

### Trisomy 21

Five studies reported on the pre-and postoperative number of patients with HAEC depending on Trisomy 21. Thirty-nine patients were suffering from both Morbus Hirschsprung and Trisomy 21, of which 12 showed preoperative HAEC (30.8%). The studies included 530 preoperative Morbus Hirschsprung patients without Trisomy 21, of which 96 showed HAEC preoperatively (18.1%). Postoperatively, 12 out of the remaining 38 (31.6%) patients in the Trisomy 21 group suffered from HAEC, whereas 128 out of 538 (23.8%) patients showed HAEC in the non-Trisomy 21 groups.

### Mortality

Fifteen of the included studies presented data on both pre-and postoperative enterocolitis-related deaths. Of these, two studies reported pre-and postoperative cases of deaths related to HAEC, three only had preoperative deaths, two only had postoperative deaths, and eight studies reported no mortality caused by HAEC. These studies include a total of 2767 preoperative patients with 403 cases of preoperative HAEC, of which 64 patients died related to HAEC (2.31% of all preoperative patients). Postoperatively, 333 of 2166 had postoperative HAEC, and 16 of those patients died from HAEC (0.74% of all postoperative patients).

### Prevalence of HAEC over time

Following the question, whether the prevalence of HAEC has changed during the past decades, all studies, excluding that only reporting on TCA, were sorted by the date of publication and divided into four groups: Group I (1979–1989); Group II (1990–1999); Group III (2000–2009); and Group IV (2010–2020). The pooled prevalence for pre-and postoperative HAEC was then calculated for each group.

The prevalence of preoperative HAEC was 0.216 for Group I (95% CI 0.135–0.309, Cochrane *Q* test, *I*^2^ = 95%), 0.176 for group II (95% CI 0.119–0.241, Cochrane *Q* test, *I*^2^ = 91%), 0.169 for group III (95% CI 0.132–0.209, Cochrane *Q* test, *I*^2^ = 82%), and 0.192 for group IV (95% CI 0.152–0.235, Cochrane *Q* test, *I*^2^ = 69%). Postoperatively, the prevalence of HAEC was 0.131 for group I (95% CI 0.103–0.161, Cochrane *Q* test, *I*^2^ = 57%), 0.157 for group II (95% CI 0.123–0.194, Cochrane *Q* test, *I*^2^ = 73%), 0.166 for group III (95% CI 0.127–0.210, Cochrane *Q* test, *I*^2^ = 84%), and 0.173 for group IV (95% CI 0.105–0.252, Cochrane *Q* test, *I*^2^ = 91%). Prevalence increased in most of the groups when considering the TCA only studies.

## Discussion

In the present study, the estimated overall prevalence of preoperatively diagnosed HAEC is 18.5%. The prevalence for preoperative HAEC is lowest in S-HD (15.2%), L-HD (17.5%) and highest in TCA (26.1%). These findings are following the published literature [13, 14]. It is well established that the risk of HAEC increases with the length of the aganglionic segment [3, 15]. The reason is mainly the absence of ganglion cells and hypertrophy of non-myelinated peripheral nerve fibres, leading to a spastic, narrowed distal bowel. The observed functional obstruction leads to faecal stasis, which is not tolerated well by patients with HD and is considered the main reason for developing HAEC. Also, there is some evidence that an altered immunological microenvironment and changes in the microbiome may contribute to the HAEC [16, 17]. This is supported by the observation that, due to the maturation of the mucosal immunity, the risk of HAEC declines during the time [18]. However, other studies provided evidence that late diagnosis of HD itself is a risk factor to develop HAEC. Two studies showed that the incidence of HAEC is lower in patients who were diagnosed with HD within the first week of life (11%) compared to those infants who were diagnosed later in life (24%) [19]. This observation has been confirmed by Lee et al., who reports an incidence of HAEC of 12% during the first week of life compared to 63% after 1 week of life [20]. Other reasons for developing HAEC are underlying genetic abnormalities, such as trisomy 21, which may have an increased risk for HAEC, at least in part from immunodeficiency [21, 22]. Data from the present study show that 30.8% of patients with trisomy 21 will at least have one episode of HAEC, and postoperatively 31.6%. Also, cardiovascular co-morbidities and lower birth weight have been linked to a higher risk of developing preoperative HAEC [23] [24]

On the other hand, pooled data suggest that the postoperative risk for developing HAEC, independently of segment length and the employed surgical technique is 18.2%, being highest in those operated with the Swenson and Duhamel approach (19.7%) and lowest with Soave approach (11.4%). However, subgroup analysis confirms no significant difference in the occurrence of postoperative enterocolitis between the three techniques, although there was a tendency towards the Duhamel approach with the highest rates in postoperative HAEC. The Duhamel technique has been traditionally used for L-HD and TCA, which may explain the relatively high rates of postoperative HAEC compared to the Soave approach. It has been shown that long-segment HD, including TCA, is a risk factor for developing postoperative HAEC [25]. However, data should be interpreted with caution since the subgroup analysis included only four studies reporting on data from S-HD and L-HD concerning pre-and postoperative HAEC when operated by the Duhamel procedure. This limits general transferability. Moreover, a direct comparison between the three major surgical procedures is biased by the difference in study selection: While the Swenson procedure could be analysed for patients with all types of lengths, the study data available for the Duhamel and Soave operations excluded some of the TCA patients or reporting only on postoperative HAEC data.

When looking into surgical techniques, whether the operations were conducted combined with an abdominal or laparoscopically assisted method compared to transanal-only, patients operated by the laparoscopically assisted approach only had the lowest rates of postoperative HAEC (10.5%), followed by a combined abdominal approach (13.4%) to transanal-only (14.5%). The reason could be that extensive transanal dissection may overstretch the sphincter and cause a partial tear, complicating the postoperative course with potentially higher rates of postoperative HAEC [26]. It is well known that the risk of postoperative HAEC is significantly increased by mechanical factors related to anastomotic complications and intestinal obstruction. Modifications such as excision of a portion of the internal sphincter or dividing the internal sphincter cuff and their numerous modifications have no significant influence on the postoperative outcome [27]. Further, the present study's data suggest that the prevalence of post-pull through HAEC is unrelated to the type of definitive surgery employed, which is also reported by other authors [25]. Although removing the aganglionic portion is curative in patients with HD, relief and proper treatment of postoperative obstruction, which potentially leads to faecal stasis, it is most important to decrease the rates of postoperative HAEC.

Furthermore, the aganglionic segment as proposed pathological aetiology of HAEC as a standalone risk factor is questionable. Other factors may contribute to postoperative HAEC. Recent studies suggested that immunological alterations in the ganglionic bowel may contribute to postoperative HAEC [28, 29]. Moreover, there seems to be some evidence that patients with HD compared to healthy individuals have a higher risk of developing IBD later in life [8]. However, up-to-date research lacks to provide common pathways to explain the pathophysiological correlation between both entities. As in preoperative HAEC, older age has been suggested as a risk factor to develop postoperative HAEC [25]. Most patients will experience an episode of HAEC within the first 18 months of life; later than that, it is rare [30]. A potential explanation could be the maturation of the intestinal immune system and increased bowel control from the patients.

Because the definition of HAEC remains imprecise based on current understanding, it makes a correct diagnosis, especially after pull-through surgery, problematic. Scoring items scales from Pastor et al. have been helpful to detect HAEC [4]. However, there is a risk of over-and underdiagnosing of HAEC. Recently, it has been proposed that a cut-off > 4 points according to the Pastor score items scale to identify suspected HAEC is more sensitive than Pastor and coworker's original cut-off > 10 points [13]. This is problematic since many patients suffer from diarrhoea postoperatively, which carries a considerable risk of overdiagnosing HAEC. Looking at the prevalence of HAEC over time by dividing the included patients into four groups showed a clear trend of an increasing prevalence of postoperative HAEC, being lowest (13.1%) in group I (1979–1989) and highest (17.3%) in group IV (2010–2020). However, there is considerable doubt if this is a natural effect or may reflect an observation bias induced due to an increased interest in HAEC in general, which is also reflected by the increased publications concerning HAEC in that period, compared to the other groups.

HAEC is a potentially life-threatening condition if left untreated. Although significantly decreased during the last decade, the mortality rate for Hirschsprung's disease still ranges between 1 and 10%. The present study's findings confirm this. In total, we found a mortality of 2.31% of all patients diagnosed with preoperative HAEC and a postoperative HAEC mortality of 0.74%. Newborns and infants seem to be more likely to experience life-threatening complications, particularly in the case of associated cardiovascular malformations [24].

Regardless of the presence of co-existing predisposing abnormalities, HAEC represented the ultimate cause of death in more than half of the patients in one series [24]. HAEC can be fatal preoperatively, between diagnosis and definitive pull-through. In contrast, a minority of patients died postoperatively. Still, the surgery proved not to increase mortality, as death is usually unrelated to surgical issues. Almost always, deaths in the postoperative period occur in patients with severe co-morbidities. To further reduce mortality preoperatively, consequent bowel management and frequent clinical reassessment are warranted. Also, for those patients at high risk, prophylactic enterostomy should be discussed to avoid HAEC. However, although enterostomies can have a protective effect over HAEC, they usually cannot prevent cardiocirculatory complications. Postoperatively, patients with severe co-morbidities should undergo strict clinical follow-up. Also, options with daily rectal irrigations could be considered [24, 31, 32].

## Strengths and limitations

A strength of the study is a large number of the included patients of the analysed original studies. A limitation is that the quality of most of the included studies was fair. Thus, the conclusions of this study should be interpreted with caution. For example, most studies failed to report both ages at operation and follow-up, defining the pre-and postoperative period. Further, the included studies show a high heterogeneity (*I*^*2*^ > 75%) and inter-study variance (*tau*^*2*^)*.* Even with this large number of studies and patients, there still was less data for the analysis of specific subgroups.

## Implications for future research

Providing accurate estimations of the long-term prognosis of patients with HD and recognising those at high risk of developing HAEC will give those patients better care, which is essential for better recovery and quality of life. Future research should involve multicenter prospective studies with standardised outcome indicators, follow-up durations and intervals, which should help design a high accuracy and transferability HAEC score. A more in-depth analysis of the prognosis of patients suffering from HAEC from the stratification of the length of aganglionosis bowel segment, age and gender and surgical procedure is also necessary.
